# Magnetoelectric/piezoelectric-based materials for coupled electrical and mechanical stimulation for bone repair: an *in silico* study

**DOI:** 10.1039/d5na00520e

**Published:** 2025-09-30

**Authors:** Ilaria Faricelli, Martina Lenzuni, Paolo Giannoni, Paolo Ravazzani, Alessandra Marrella

**Affiliations:** a Institute of Electronics, Computer and Telecommunication Engineering (IEIIT), National Research Council of Italy (CNR) Genoa Italy martina.lenzuni@cnr.it; b Department of Informatics, Bioengineering, Robotics and Systems Engineering (DIBRIS), University of Genoa Genoa Italy; c Department of Experimental Medicine, Biology Section, University of Genoa Genoa Italy; d Institute of Electronics, Computer and Telecommunication Engineering (IEIIT), National Research Council of Italy (CNR) Milan Italy

## Abstract

Bone repair is a complex process that requires the simultaneous presence of mechanical and electrical signals to replicate the physiological communication between acting forces, bone, and nerve cells. In this work, a new approach for bone repair is proposed that combines the properties of magnetoelectric nanoparticles (MENPs) and the piezoelectric properties of hydroxyapatite (HAP) particle to provide coupled mechanical and electrical stimulation. Although HAP is widely used in biological applications, its piezoelectric properties have never been modelled within a multiphysics framework. The modelling herewith proposed focuses on the magnetoelectric response of MENPs embedded within an alginate hydrogel matrix subjected to a DC magnetic field, on the effect of MENP concentration on the resulting electric field distribution, and on the mechanical stress generated by a single HAP particle in response to the electric field elicited by MENPs. A final 3D model is developed to investigate the coupled effects of electrical and mechanical stimulation on a human cell. The results show that the electric field generated throughout the alginate hydrogel matrix reaches values known to upregulate key markers associated with extracellular matrix mineralization. Moreover, a single MENP-activated HAP particle induces a localized von Mises stress of up to 4.91 N m^−2^, able to trigger osteogenic processes, such as osteoblast proliferation and differentiation.

## Abstract 2

Bone repair is a complex process that requires the simultaneous presence of mechanical and electrical signals to replicate the physiological communication between acting forces, bone, and nerve cells.

## Graphical abstract

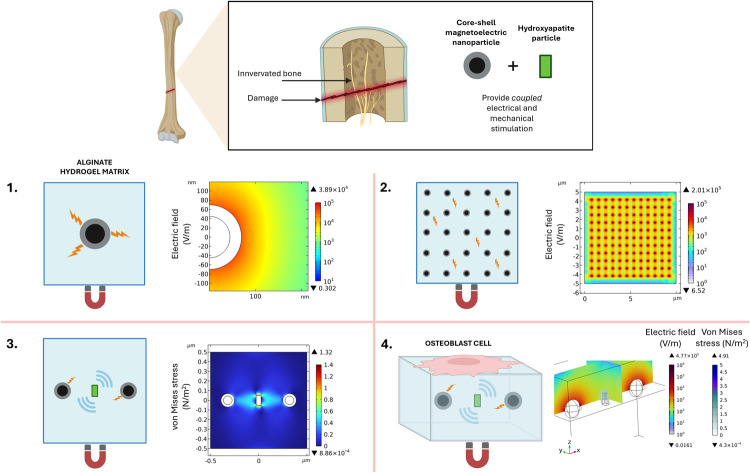

## 1 Introduction

Bone development and regeneration rely on the complex interplay of factors within the bone microenvironment, which include the vascular, immune and nervous systems.^1–3^ In particular, nerve fibers are located within the trabecular bone, periosteum, and the callus formed during the fracture healing process. Recent evidence highlights the key role of sensory nerves in bone repair, as they innervate bone tissue and regulate its metabolism through the release of neurotransmitters, neurotrophins, and neuropeptides acting as bone-neuro mediators. Moreover, the harmonious crosstalk between bone and nerves is evidenced by the expression of the related receptors in cells of the nervous system as well as bone lineage cells.^2^

Traditional scaffolds, designed to selectively trigger bone repair, have failed to provide the specific signals involved in the complex neuro-osteogenic interplay. Recently, several approaches have been gradually explored to provide a harmonious neurogenesis and osteogenesis. The first attempted strategy consisted of designing grafts embedded with neurotrophic factors in order to reproduce the physiological communication between bone and nerves. For example, orthopedic implants containing biodegradable magnesium have been adopted due to the positive role of magnesium, which stimulates the secretion of calcitonin gene-related peptides (CGRP) from sensory nerves, indirectly promoting bone repair.^4^ In another work, nerve growth factor (NGF) was encapsulated in a collagen-based scaffold in order to stimulate neurogenesis; this approach resulted in enhanced bone formation relative to the control group, offering evidence of the indirect stimulation of bone repair through the promotion of neurogenesis.^5^

However, these approaches do not consider the physical external stimuli to which the bone microenvironment is subjected. In particular, it is known that physical stimulation, including tensile and compressive stresses, can promote bone regeneration; cells can detect external mechanical stimulation and transduce it into biochemical signals through specific mechanoreceptors.^6^ Furthermore, the bone microenvironment exhibits distinct electrical properties, and it has been demonstrated that the application of electrical stimuli promotes osteogenic differentiation and stimulates nerve growth.^7^ Nevertheless, these approaches usually necessitate the direct placement of electrodes (for electrical stimulation) or tensile devices (for mechanical stimulation) at the treatment site, making them highly invasive and impairing the delivery of the stimuli with high specificity. Therefore, the design of smart scaffolds capable of wirelessly providing cells a full-range of stimulations is of growing interest.

In this context, magnetoelectric nanoparticles (MENPs) capable of generating electric fields when activated by external magnetic stimulation are emerging in different biotechnology fields.^8–10^ The most commonly used configuration of MENPs is the core–shell CoFe_2_O_4_ (cobalt ferrite, “CFO”)–BaTiO_3_ (barium titanate, “BTO”) system, which combines the ferromagnetism of CFO, acting as the core, with the electrical polarization and piezoelectric properties of BTO, the shell. However, their application in bone tissue engineering is still limited,^11^ requiring specific biomimetic chemical and mechanical cues to promote osteogenesis.^2,12^

To bridge this gap, the combination of MENPs with piezoelectric materials, such as hydroxyapatite (HAP), offers an innovative solution. HAP is a well-established material in bone tissue engineering due to its chemical and structural similarity to natural bone minerals.^13,14^ Beyond its biocompatibility, HAP also exhibits piezoelectric properties similar to those of bone tissue, which enable the transduction of an electric signal to a mechanical stimulus. In order to fully mimic the coupled mechanical–electrical signals present in bone, a composite alginate hydrogel-based matrix embedded with MENPs and a HAP particle has been designed, in this study, to provide such double stimulation. MENPs, when properly activated by an external magnetic stimulator, generate a localized electric field that dissipates within a conductive water-based matrix. The electric current is then detected by the HAP particle, which convert the signal into a mechanical force. At the same time, it is also well known that the delivery of electrical stimuli to bone-resident nerve cells enhances their release of pro-osteogenic factors, generating a self-sustaining cross-signaling network that ultimately implements bone regeneration. The spatial directions of bone development follow gradients of neuropeptides and signals that are released by nervous fibers; moreover, this orchestrated signaling system takes places not only during skeletal development, but is also resumed during bone repair events: areas of bone with the highest metabolic activity receive the richest sensory and sympathetic innervation and resident bone precursor cells here express receptors for many of the neuronal messengers. In this light, MENPs can be seen as double-acting stimuli transducers, which may provide local resident nerve cells with the proper signals to enhance the release of neuropeptides, such as CGRP, involved in bone remodeling and repair,^15^ as well as mechanical stimuli to the piezoelectric-responsive HAP components of the construct. In order to predict the optimal setting, the goal of our study was to conduct an *in silico* assessment of the effects of MENP concentrations, HAP particle size, and HAP particle orientation on the resulting electrical and mechanical outputs. The stimuli received by a cell placed over the alginate hydrogel matrix were also modelled, with the predicted values generated by our model aimed at providing insights for the fabrication of effective mechanically- and electrically-coupled bioactive substrates.

## 2 Materials and methods

COMSOL Multiphysics® 6.1 was adopted to develop a series of computational models, each designed to capture distinct and complementary aspects of the composite's behavior. Specifically, the simulations were performed to model (i) the magnetoelectric response of an individual MENP embedded within an alginate hydrogel-based matrix under a DC magnetic field, (ii) the collective interactions and electric field distributions arising from different MENP concentrations within the alginate hydrogel matrix, (iii) the complex interplay between MENPs and a HAP particle, elucidating their synergistic effects on local electric field and stress generation, and (iv) a 3D model incorporating MENPs, a HAP particle, and a human cell to investigate the multifaceted interactions between electrical stimulation and stress generation under a controlled DC field.

### 2.1 MENP modelling within an alginate hydrogel matrix

**Fig. 1:**
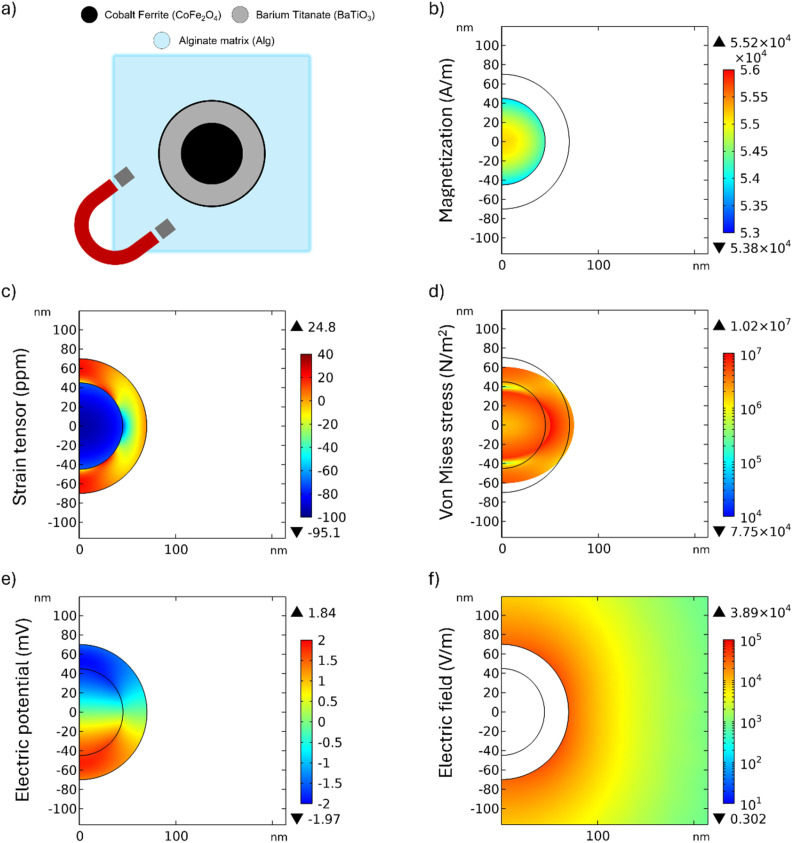
Behavior of a single MENP in an alginate hydrogel matrix under stimulation by a DC magnetic field of 300 mT. (a) Schematic representation of the MENP–alginate gel system geometry; (b) core magnetization *M* (A m^−1^); (c–e) strain *ε* (ppm), von Mises stress *σ* (N m^−2^), and electric potential *V* (mV) distribution generated in the MENP; (f) electric field *E* (V m^−1^) distribution in the surrounding alginate hydrogel matrix.

To investigate the (i) behaviour of a single MENP within an alginate hydrogel matrix under a DC field of 300 mT (applied along the boundaries of the medium in the *z*-direction), an axisymmetric 2D model was developed. The applied field strength of 300 mT has been widely used in the literature for both *in silico* and *in vitro* studies involving magnetic fields.^9,16^ As reported in Fig. 1(a), the model comprises 3 distinct domains: (i) the MENP core, (ii) the MENP shell and (iii) the surrounding alginate hydrogel matrix. The MENP was represented as a core–shell nanoparticle with magnetoelectric properties. The core was composed of a magnetostrictive material (cobalt ferrite, CoFe_2_O_4_, “CFO”) with a radius of 45 nm, while the shell was composed of a piezoelectric material (barium titanate, BaTiO_3_, “BTO”) with a thickness of 25 nm. The alginate hydrogel matrix, where the MENP is embedded, was represented as a homogeneous and isotropic material and modelled as a square domain with a 1 μm side.

The material properties were obtained from the literature, the COMSOL built-in library (https://www.comsol.com), and data sheets provided by the MENP manufacturer (https://www.nanoshel.com/). A summary of the material parameters is provided in Table S1.

To model the magnetoelectric response of the MENPs, a multiphysics approach was implemented, integrating the magnetic fields, solid mechanics, and electrostatics modules within the COMSOL environment, as reported in our previous studies.^17,18^ Briefly, the magnetostriction multiphysics interface was used to couple the magnetic fields and solid mechanics modules in the core domain, while the piezoelectricity multiphysics interface was employed to couple the solid mechanics and electrostatics modules in the shell domain.

To quantify the magnetoelectric behaviour of MENPs, the magnetoelectric coefficient *α*_ME_ was estimated according to the methods previously reported in the literature. In particular, it was calculated as (1) the ratio between the average electric field intensity at the MENP outer border and the external magnetic field intensity, and (2) the ratio between the electric potential difference across the MENP shell and the external magnetic field intensity multiplied by the MENP diameter.^19,20^

In a second study, different concentrations of MENPs within the alginate hydrogel matrix were subjected to a static magnetic field of 300 mT along the *z*-axis and a two-dimensional (2D) model was developed to represent the distribution of MENPs within the same alginate domain. Each MENP was modelled as an individual dipole, with its electric potential derived from the numerical results obtained in the previous computational study.

Here, the alginate hydrogel matrix was modelled as a square domain of 10 μm side. The simulations were performed for MENP concentrations of 0.5%, 1%, 1.5%, 2%, 2.5% and 3%, where the concentration was defined as the ratio between the total area covered by the MENPs and the alginate hydrogel area, expressed as a percentage. This definition represents a simplification based on the 2D model; the corresponding 3D volume fraction was estimated by extending the same particle arrangement to a 10 × 10 × 10 μm^3^ volume, calculating the volume fraction as the percentage of the gel volume occupied by the MENPs. A comparison between a uniform and random MENP distribution at a coverage area of 2% was also performed to evaluate the effect of spatial distribution on electric field generation under magnetic stimulation. All MENPs were assumed to be uniformly aligned in the direction of the external magnetic field.

### 2.2 HAP particle modelling

The HAP particle was modeled as a piezoelectric material with lengths of 100 nm, 200 nm, and 400 nm, while maintaining a constant width of 50 nm. These dimensions were selected on the basis of their prevalence in the literature,^21^ despite the wide range of possible HAP particle structures. The 2D rectangular model represents the projected view of the characteristic 3D hexagonal structure of hydroxyapatite.^22^ All material properties were sourced from the literature and are detailed in Table S1. For the 3D model, the HAP particle was represented as a piezoelectric rod-like structure with a hexagonal base (50 nm edge-to-edge diameter) and a length of 100 nm, in accordance with previous studies.^23^ Within the COMSOL environment, the piezoelectricity multiphysics interface was used to couple the solid mechanics and electrostatics modules in the HAP particle domain. In fact, due to its piezoelectric properties, when subjected to an external electric field, the HAP particle undergoes mechanical deformation, inducing localized stress in its surrounding alginate hydrogel matrix. The piezoelectric effect governing this response is described by the following constitutive equations in the stress–charge form:

1 *σ* = *c*_E_*ε* − *e*^T^*E*

2 *D* = *eε* + *ε*_0_*ε*_rs_*E*

where *ε*_0_ is the vacuum permittivity, *σ* is the stress, *ε* is the strain, *E* is the electric field, and *D* is the electric displacement field. The material parameters *c*_E_, *e* and *ε*_rs_ correspond to the material elasticity matrix, the coupling matrix and the relative permittivity. Strain (*ε*) is defined as the ratio between the displacement (Δ*L*) and the original length (*L*), according to the following equation:

3 *ε* = Δ*L*/*L*

### 2.3 Interactions between MENPs and a HAP particle

To study the interaction between MENPs and a HAP particle under a DC field, 2D models were first developed. Each geometrical model was composed of two MENPs and a HAP particle embedded within the alginate hydrogel matrix, which was designed as a square domain of 1 μm side. The inter-particle distance between MENPs was set according to the conditions corresponding to a 3% MENP concentration (Table 1). The MENPs were modelled as individual electric dipoles, with the electric potential assigned according to the results of previous simulations, as detailed in the preceding sections.

**Table 1:** Percentages of alginate hydrogel matrix area covered by various electric field (*E*) thresholds for each MENP concentration, corresponding estimated 3D volume fraction and relative MENPs distance

Estimated MENPs volume fraction (%)	MENPs area coverage (%)	MENPs distance (μm)	Electric field threshold (V m^−1^)				
			*E* > 10	*E* > 10^2^	*E* > 10^3^	*E* > 10^4^	*E* > 10^5^
0.04	0.5	1.40	99.88%	98.26%	77.06%	30.91%	0.80%
0.1	1.0	1.00	99.91%	99.29%	83.68%	33.35%	0.86%
0.16	1.5	0.90	99.98%	99.40%	88.21%	35.05%	0.90%
0.23	2.0	0.80	99.98%	99.58%	91.32%	36.52%	0.93%
0.34	2.5	0.70	99.99%	99.66%	92.96%	38.34%	0.97%
0.42	3.0	0.65	99.99%	99.74%	94.62%	39.83%	1.00%

To model the interaction between MENPs, a HAP particle and the alginate hydrogel matrix, the physics modules electrostatics, electric currents and solid mechanics were used. This integrated framework enabled the simultaneous simulation of (i) the electric field generated by MENPs and its spatial distribution near the HAP particle, (ii) the piezoelectric response of the HAP particle under the induced electric field generated by MENPs subjected to an external DC magnetic field, and (iii) the mechanical stress exerted by the HAP particle on the surrounding alginate hydrogel matrix.

In a subsequent analysis, to improve the reliability and predictive capability of the model, the interactions between the aforementioned particles and the surrounding environments were studied in a three-dimensional (3D) model. In particular, HAP and MENPs were modelled as fully embedded in the alginate hydrogel matrix or placed at the interface between the alginate hydrogel matrix and a cell ideally cultured over the gel. The design of MENPs and a HAP particle and their relative positioning were set in the model as described above. The human cell parameters, representative of an osteoblast cell, were derived from the literature and are reported in Table S1.^24–26^ This simplified model with homogeneous properties was used in the present computational studies to capture the essential electromechanical behaviour of these cells. The physics modules electrostatics, electric currents and solid mechanics were used, together with the piezoelectricity multiphysics coupling that couples electrostatics and solid mechanics elements, to mimic and model the interaction between the MENPs, the HAP particle, the hydrogel, and a human cell, as previously indicated. This approach enabled the simultaneous derivation of (i) the electric field experienced by the HAP particle, and (ii) the mechanical stress exerted by the HAP particle on the surrounding environments, including the alginate hydrogel matrix and adjacent cell.

## 3 Results and discussion

### 3.1 MENP behavior in an alginate hydrogel matrix

The magnetization of the core of a single MENP, under the effect of an external static magnetic field of 300 mT, reaches the maximum value of 5.52 × 10^4^ A m^−1^, as depicted in Fig. 1(b). The strain generated in the core, due to its magnetostrictive nature, propagates into the piezoelectric shell as is shown in Fig. 1(c). Fig. 1(d) shows the von Mises stress distribution, with the maximum value (1.02 × 10^7^ N m^−2^) localized at the core–shell interface. Given the piezoelectric nature of the shell, the mechanical stress induces the MENP polarization and generation of an electric potential up to ±1.36 mV on the surface of the shell, with the maximum value found at the interface between the core and the shell domain, as shown in Fig. 1(e). This potential difference produces a localized electric field, transforming MENPs into “nanoelectrodes”, potentially capable of generating electrical stimulation at the cellular level without the need for externally applied electrodes. Fig. 1(f) evidences the distribution of the electric field generated by the MENP in the surrounding alginate hydrogel matrix. From the shell surface of the MENP to a distance of 400 nm into the alginate hydrogel matrix, the electric field varies between 10^4^ and 10^2^ V m^−1^, as shown in Fig. S1(a), (b) and 1(f). The value of the magnetoelectric coefficient, estimated as the ratio between the average electric field strength at the outer edge of the MENP and the applied external magnetic field strength, was 0.11 V cm^−1^ Oe^−1^. This value is consistent with that obtained as the ratio between the electric potential difference across the MENP shell and the product of the magnetic field strength and the particle diameter, yielding 0.07 V cm^−1^ Oe^−1^. Both methods produced comparable results, which are in good agreement with values reported in the literature for similar particles.^17,27^

**Fig. 2:**
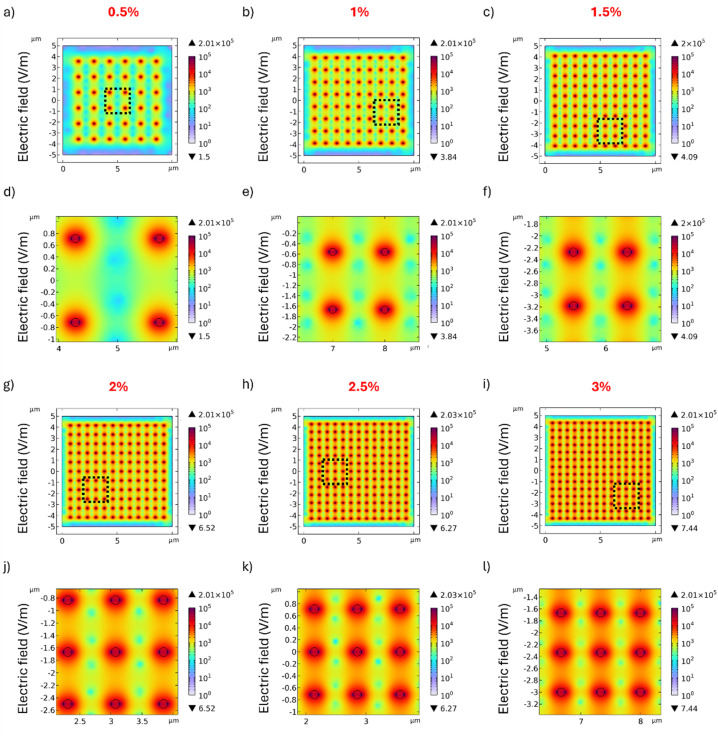
Effects of MENP concentration on the electric field distribution. Electric field distributions are generated by different MENP concentrations in the alginate hydrogel matrix upon DC field activation. MENP concentrations of 0.5%, 1%, 1.5%, 2%, 2.5% and 3% are shown in (a–c) and (g–i), respectively. (d–f) and (j–l) represent a zoomed-in view of a 2 μm × 2 μm area within each corresponding region (delimited by dotted lines), providing a more detailed view of the electric field distribution.

Fig. 2 illustrates the electric field distribution within the alginate hydrogel matrix containing different concentrations of MENPs, uniformly distributed, under the application of a DC magnetic field of 300 mT. Specifically, Fig. 2(a) shows the distribution at 0.5%, with the corresponding zoomed-in view in Fig. 2(d). Similarly, Fig. 2(b) and (e) report the case for 1%, Fig. 2(c) and (f) for 1.5%, Fig. 2(g) and (j) for 2%, Fig. 2(h) and (k) for 2.5%, and Fig. 2(i) and (l) for 3%. For each concentration, the distance between two consecutive MENPs within the alginate hydrogel matrix is shown in Table 1. Despite variations in the spatial configuration of the particles, the maximum value of the electric field remains almost unchanged for all concentrations considered and is localized on the shell of each MENP, where it depends mainly on the magnetization of the individual particle, with minimal influence from the surrounding environment. On the other hand, the percentage of the alginate hydrogel matrix area covered by electric field values above different thresholds increases with increasing MENP concentration, as shown in Table 1. To investigate whether this trend was affected by the MENPs′ spatial arrangement and to simulate a more realistic situation, the MENPs at 2% concentration were also randomly distributed (Fig. S2). The percentages of alginate hydrogel matrix area covered by different electric field thresholds are shown in Table S2. The results for the randomly distributed MENPs are extremely close to those observed for the uniform distribution.

The maximum electric field value slightly increases as some MENPs end up closer together, while the minimum electric field value decreases due to larger inter-particle gaps.

The observed electric field distributions hold significant implications for biomedical applications, particularly in tissue engineering and regenerative medicine. Previous studies have established that specific electric field strengths can modulate cellular behavior, influencing processes such as proliferation, differentiation, and extracellular matrix synthesis. In the context of innervated bone tissue, multiple cell types, including Schwann cells and osteoblasts, are known to be responsive to electric fields.^28^ Schwann cells, essential for nerve regeneration, exhibit enhanced proliferation rates and increased secretion of neurotrophic factors such as nerve growth factor (NGF) and brain-derived neurotrophic factor (BDNF) when exposed to electric fields exceeding 100 V m^−1^.^29^ These findings have significant implications for peripheral nerve repair and neuromodulation strategies. Moreover, recent evidence suggests that these factors may play a direct role in regulating the process of bone regeneration.^30^ Similarly, human mesenchymal stem cells (hMSCs) exposed to an electric field of 100 V m^−1^ demonstrate accelerated early osteogenic differentiation.^31^ Furthermore, osteoblast activity and extracellular matrix remodelling are significantly influenced by electric field strength. An electric field of 200 V m^−1^ has been shown to enhance osteoblast proliferation and up-regulate key markers associated with matrix mineralization.^32^

### 3.2 2D interactions between MENPs and a HAP particle

**Fig. 3:**
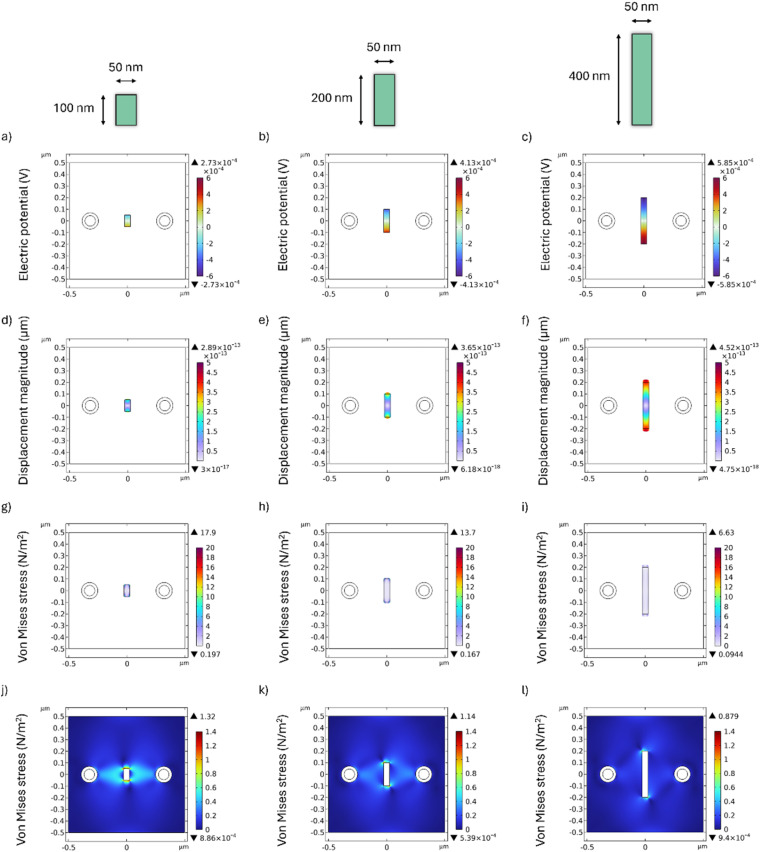
Behavior of different HAP particles placed between two MENPs in an alginate hydrogel matrix under a DC field. (a–c) The electric potential established on the HAP particle of 50 nm in width and 100, 200 and 400 nm in length, respectively. The corresponding displacement is shown in (d–f), and the von Mises stress on the HAP particles is shown in (g–i). (j–l) indicate the von Mises stress generated on the alginate hydrogel matrix.

The electric field generated by MENPs within the alginate hydrogel matrix induces polarization in the HAP particle domain. The spatial distribution of the electric potential established within the HAP particle, as a function of particle dimensions, is shown in Fig. 3(a)–(c). Fig. 3(a)–(c) correspond to small, medium, and long HAP particles, respectively. The longer particle (Fig. 3(c)) shows a more intense polarization field, likely attributed to its larger surface area, which allows more piezoelectric material to interact with the electric field generated by the MENPs.^33^ This induced polarization generates mechanical forces within the HAP particles, resulting in observable displacements from their initial positions, as shown in Fig. 3(d)–(f). Fig. 3(d)–(f) correspond to small, medium, and long HAP particles, respectively. Fig. S3 depicts the strain undergone by HAP particle of different lengths, showing that smaller particles undergo greater strain. This occurs since the displacement-length ratio, described by eqn (3), is greater for shorter particles. Also, according to the piezoelectric equation (eqn (1)), greater strain corresponds to greater stress. Indeed, Fig. 3(g)–(i) highlights this phenomenon, showing that the von Mises stress within the HAP particle reaches a maximum value of 17.9 N m^−2^ for the smallest particle (100 nm in length, Fig. 3(g)) while it decreases to 13.7 N m^−2^ for the 200 nm particle (Fig. 3(h)) and to 6.63 N m^−2^ for the 400 nm particle (Fig. 3(i)). This internal stress also influences the surrounding alginate hydrogel matrix. The mechanical stress exerted by the HAP particle on the alginate hydrogel matrix is shown in Fig. 3(j)–(l). Fig. 3(j)–(l) correspond to small, medium, and long HAP particles, respectively. The results show that the highest maximum value, 1.32 N m^−2^, is achieved by the smallest particle with a length of 100 nm and a width of 50 nm (Fig. 3(j)). As expected, the higher stress concentration within the smaller particle results in higher stress applied to the alginate hydrogel matrix. Conversely, larger particles distribute stress over a larger volume, thereby reducing the stress magnitude transmitted to the alginate hydrogel matrix, with values of 1.14 N m^−2^ for the 200 nm particle (Fig. 3(k)) and 0.88 N m^−2^ for the 400 nm particle (Fig. 3(l)). Furthermore, it is important to note that the properties of the alginate hydrogel matrix, as an energy-dissipating matrix, modulate the distribution of stress generated by the HAP particle. According to Hooke's law,^34^ the elastic constants that define the stress–strain relationship are directly determined by Young's modulus and Poisson's ratio. In our simulations, physiologically relevant values of these parameters were assigned (Table S1), representative of soft, biocompatible hydrogels commonly used in tissue engineering applications.^35–39^ It should be underlined that the electric potential on the HAP particle varies depending on its orientation due to the different interactions with the electric field generated by MENPs. In fact, depending on the orientation of the HAP particle, different components of the electric field will act differently on it, inducing a variable potential (Fig. S4(a) and (b)). HAP has a hexagonal crystal structure with anisotropic piezoelectric properties. For this reason, the piezoelectric coefficients have different values depending on the crystallographic direction. Its strongest piezoelectric coefficient is generally observed along the *c*-axis (*d*_33_), while other coefficients, such as *d*_31_, show lower values.^40^ This anisotropy explains why the orientation of the particles relative to the field direction strongly influences the resulting piezoelectric response. Similarly, the displacement of the HAP particle is highly dependent on its orientation, as the particle's alignment alters the interaction between the electric field components and the piezoelectric tensor.^41,42^ When the particle is perpendicular to the field (*i.e.*, placed horizontally) (Fig. S4(c)), the displacement occurs primarily along its longitudinal axis, resulting in a lower strain. In contrast, when the particle is partially rotated (Fig. S4(d)), additional electric field components act on the HAP particle, enhancing the induced displacement. The same trend is observed for the von Mises stress, which reaches its highest values in the rotated particle (Fig. S4(d)), with an average stress value of 7.45 N m^−2^ intrinsic to the HAP particle, followed by the horizontally oriented particle with 4.67 N m^−2^ (perpendicular to the MENP dipole) and finally the vertically oriented particle (parallel with the MENP dipole) with 2.83 N m^−2^. It is crucial to emphasize that the average stress value across the particle should be considered, as we did, rather than solely relying on the maximum von Mises stress, to ensure a more representative and accurate evaluation of the material's mechanical response.

### 3.3 3D interactions between MENPs and a HAP particle

**Fig. 4:**
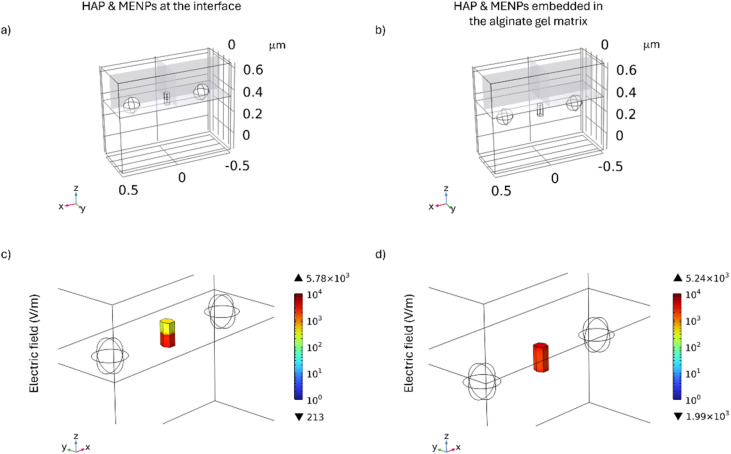
Three-dimensional spatial distribution and electric field distribution of two configurations involving MENPs, HAP particles, the alginate hydrogel matrix, and a human cell. In (a), the HAP particle and MENPs are placed at the interface between the alginate hydrogel matrix and a human cell, whereas in (b), they are fully embedded within the alginate hydrogel matrix. Both configurations highlight the *XY*, *YZ* and *ZX* planes on the cell domain. (c) and (d) represent the corresponding electric field distribution (V m^−1^) on the HAP particles for each configuration when the HAP particle is placed between two MENPs under a DC field.

Based on the results presented in Fig. 3, the smallest HAP particle, measuring 50 nm in width and 100 nm in length, induces the highest von Mises stress within the alginate hydrogel matrix. Consequently, this specific particle size was selected for subsequent 3D analyses to investigate the distribution of stress exerted on both the alginate hydrogel matrix and a human cell. To ensure a conservative assessment, two distinct configurations were considered, as illustrated in Fig. 4. In the first configuration (Fig. 4(a)), the particles are positioned at the interface between the alginate hydrogel matrix and the cell, whereas in the second configuration (Fig. 4(b)), they are entirely embedded within the alginate hydrogel matrix. The distance between the top surface of the HAP particle and the interface between the alginate hydrogel matrix and the cell is 0.05 μm. Given that the relative positioning of the HAP particles is expected to be random, the analysis was conducted under the “worst-case scenario” by considering the configuration in which the HAP particle remains unrotated along the *z*-axis, maintaining a vertical orientation within the system. This choice was made as it corresponds to the condition that minimizes the von Mises stress exerted by the HAP particle on the alginate hydrogel matrix, while other orientations could potentially generate even higher stress levels. In an experimental scenario, HAP particles would tend to distribute around the cell, enabling mechanical stress to be transferred directly to the cell when the particles are in direct contact, or partially dissipated through the alginate hydrogel matrix when the particles are in close proximity. Although the hydrogel is a soft matrix, it could still serve as an effective medium for transmitting the mechanical forces generated, thus facilitating localized nano-mechanotransduction at the cellular level.^43,44^ The electric field distribution on the HAP particle placed at the interface between the alginate hydrogel matrix and the cell is shown in Fig. 4(c). In this configuration, the electric field distribution on the surface of the HAP particle is not uniform, but varies depending on whether the surrounding material is the alginate hydrogel matrix or the cell. Specifically, the portion of the HAP immersed in the alginate hydrogel matrix reaches a maximum electric field value of 5.83 × 10^3^ V m^−1^, while the portion immersed in the cell reaches 2.28 × 10^3^ V m^−1^. Overall, the average electric field across the HAP particle domain is found to be 2.04 × 10^3^ V m^−1^. In contrast, when particles are fully embedded in the alginate hydrogel matrix, the electric field distribution is more uniform with respect to the previous configuration, as shown in Fig. 4(d). In this case, a maximum electric field value of 5.24 × 10^3^ V m^−1^ is reached, and the average electric field across the HAP particle domain is found to be 2.87 × 10^3^ V m^−1^. These results are consistent with the electric current constitutive relationship, as derived from Ohm's law and its links to conductivity and to the electric displacement field.

**Fig. 5:**
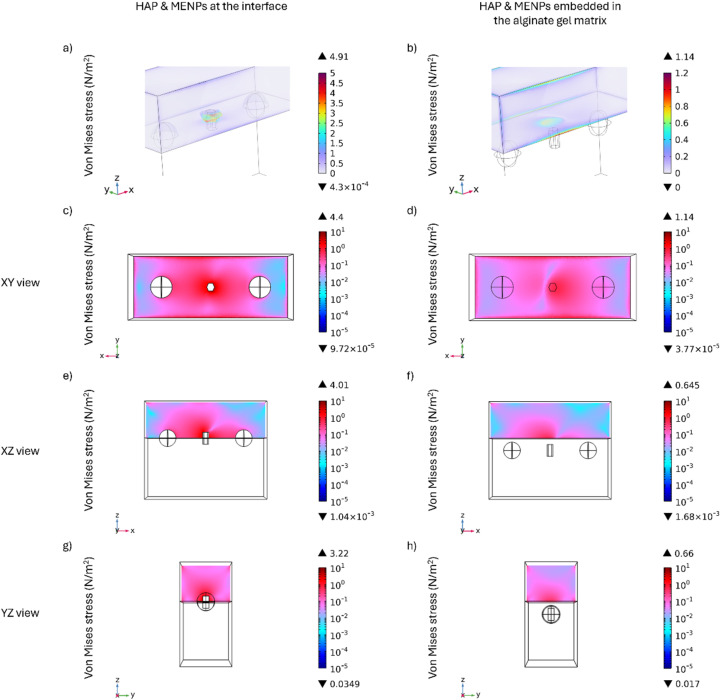
3D distribution of the von Mises stress on the alginate hydrogel matrix and a human cell, exerted by a single HAP particle placed between 2 MENPs under a DC field. (a) The von Mises stress exerted by a HAP particle on the cell when HAP particle and MENPs are located at the interface between the alginate hydrogel matrix and the cell, and (b) the same stress when both are immersed in the alginate hyrdrogel matrix. The respective projections are shown in (c) and (d) for the *XY*-plane, (e) and (f) for the *XZ*-plane, and (g) and (h) for the *YZ*-plane, respectively. The *XY*-plane was taken at the interface between the alginate hydrogel matrix and the human cell, while the *XZ*-plane and the *YZ*-plane were taken at the middle of the HAP particle.

As shown in Fig. 5, the distribution of von Mises stress induced by the HAP particle differs between the two analysed configurations. In particular, when the HAP particle is at the interface between the cell and the alginate hydrogel matrix, the maximum value of the stress on the cell domain reaches 4.91 N m^−2^ (Fig. 5(a)), the highest of all configurations. In the configuration where the HAP particle is immersed within the alginate hydrogel matrix, the maximum stress value on the cell domain reaches 1.14 N m^−2^ (Fig. 5(b)).

Taking these findings together, these results are promising when compared with experimental data. In particular, in the first configuration, the induced stress exceeds the 2 N m^−2^ threshold, which has been shown to be sufficient to stimulate the proliferation and differentiation of human osteoblasts *in vitro*, leading to the activation of intracellular signaling pathways and the up-regulation of proteins involved in cell adhesion.^45^ Moreover, previous studies have demonstrated that an external stress of 3 N m^−2^ induces osteocyte membrane deformation, triggering ATP release and initiating a cascade of biological processes involved in the mechanotransduction and bone repair.^46^ Additionally, mechanical stimulation at a lower stress level of 1 N m^−2^ has been reported to enhance bone formation by decreasing the RANKL/OPG ratio in murine long bone osteocyte Y4 cells, thereby influencing bone remodelling processes.^47^

It is important to highlight that the stress values obtained from the modelling studies are derived from the behavior of a single HAP particle, whose nanometric dimensions enable a highly localized stress distribution in the surrounding environment. Specifically, the analysis considers the scenario in which the smaller HAP particle (100 nm in length) remains unrotated along the *z*-axis, maintaining a vertical orientation within the system. This configuration is regarded as the most conservative, as alternative orientations resulted in higher stress values. Moreover, as the study examines a single nanoparticle, the mechanical stress remains confined to a limited region of the cell. However, in a realistic scenario, multiple HAP particles may be distributed across the entire interface between the gel and the cell, leading to mechanical stress being induced at multiple sites. This suggests that the overall mechanical stimulation could be significantly enhanced in the presence of multiple interacting particles.

**Fig. 6:**
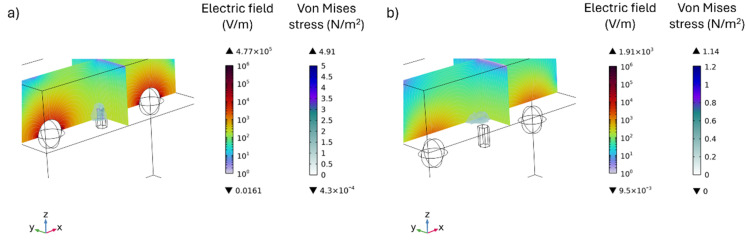
Combined 3D distribution of the electric field and stress on the human cell produced by a single HAP particle placed between two MENPs under a DC field. (a) The coupled electromechanical effect for the configuration in which the HAP particle and the MENPs are at the interface between the alginate hydrogel matrix and the cell, and (b) the configuration in which the particles are fully embedded in the alginate hydrogel matrix.

Lastly, as shown in Fig. 6, the combined 3D distribution of the electric field and the resulting von Mises stress corroborates the presence of a coupled electromechanical effect produced by the interaction between MENPs and HAP particle. These results demonstrate the potential for a synergistic action between the two systems, which is particularly significant in the context of bone tissue engineering, where both electrical and mechanical stimuli play a key role in supporting bone remodelling processes.

## 4 Limitations and future work

This study introduces, for the first time, the concept of coupling magnetoelectric core–shell nanoparticles with the piezoelectricity of hydroxyapatite for a coupled electrical/mechanical stimulation. Although innovative, the present work remains theoretical, and some limitations need to be considered.

First, the core–shell MENP and HAP models were modelled while neglecting possible morphological irregularities, size polydispersity, and structural defects typically observed in real nanoparticles.^48^ In addition, the simulations were conducted under the assumption of a static and uniform DC magnetic field, whereas experimental applications will involve the application of dynamic magnetic fields (DC + AC), by means of specific coils. Finally, the influence of the biological physiological environment – such as the presence of a fluid environment and the formation of a protein corona due to protein adsorption on the nanoparticle surface – was not included in the model, and *in vitro* experiments are still under investigation. Future research will aim to address these aspects by extending the computational model to more realistic geometries and orientations of MENPs and HAPs, and by incorporating the anisotropic response of HAP under complex tissue-mimicking conditions. More advanced and realistic 3D cell models will be developed, together with the inclusion of multiple HAP particles randomly dispersed within the system. A more accurate representation would also consider the different cell compartments, such as the cell membrane and the cytoplasm, assigning different electrical and mechanical properties to each. Moreover, coupling computational predictions with *in vitro* experiments will be crucial to validate the model and to assess the real potential of MENPs in modulating HAP activity for neuro-bone tissue engineering. Furthermore, comparative *in vitro* studies with piezoelectric stimulation approaches currently established in the biomedical field, such as ultrasound,^49^ could evaluate the relative effectiveness and advantages of strategies based on external magnetic fields, which are transparent and intrinsically safe for the body.

## 5 Conclusions

The combination of mechanical and electrical stimulation is essential for replicating the physiological communication between bone and nerve cells. In this *in silico* study, a novel approach for bone repair is proposed by leveraging the magnetoelectric properties of MENPs and the piezoelectricity of well-established HAP particles in bone tissue engineering. Here, the electric field distribution generated by single and multiple MENPs (with a diameter of 140 nm) embedded in an alginate hydrogel matrix was derived through a multiphysics modelling framework, providing insights into the spatial distribution of the electric field within the system. Subsequently, by positioning a single HAP particle between two MENPs, the optimal size and spatial configuration were identified to maximize the piezoelectric response, upon MENP-mediated electrical activation. A further analysis was performed to assess the resulting electric and mechanical stimuli acting on a human cell in contact with the composite substrate. The simulations indicate that (i) the electric field generated by MENPs reaches values known to enhance osteoblast proliferation and up-regulate key markers associated with extracellular matrix mineralization; (ii) the mechanical stress (von Mises stress) exerted by a single HAP particle (50 nm in width and 100 nm in length, exhibiting a rod-like structure) on a human cell is on the order of few N m^−2^, enough to stimulate osteogenic processes. Moreover, since a single particle induces localized mechanical stress, a broader distribution of HAP particles across the cell could result in mechanical stimulation at multiple sites, further enhancing the regenerative potential of the proposed composite. This integrated approach, combining magnetoelectricity and piezoelectricity, holds significant potential for advancing bone repair strategies, providing insights for the fabrication of effective mechanically- and electrically-coupled bioactive substrates.

## Author contributions

I. F.: methodology, software, formal analysis, writing – original draft, visualization; M. L.: methodology, software, formal analysis, writing – original draft; P. G.: conceptualization, resources, data curation, writing – review & editing, supervision; P. R.: resources, writing – review & editing, supervision, funding acquisition, project administration; A. M.: conceptualization, methodology, formal analysis, writing – review & editing, supervision, funding acquisition, project administration.

## Conflicts of interest

There are no conflicts to declare.

## Supplementary Material

NA-OLF-D5NA00520E-s001

## Data Availability

The data supporting this article have been included as part of the supplementary information (SI). Supplementary information is available. See DOI: https://doi.org/10.1039/d5na00520e.
