# Supplementary material for: Magnetoelectric/piezoelectric-based materials for coupled electrical and mechanical stimulation for bone repair: an in silico study
Source: Nanoscale Adv. 2025 Sep 30;7(23):7768–79. doi: 10.1039/d5na00520e (PMC12536647; doi:10.1039/d5na00520e)
Supplement: NA-007-D5NA00520E-s001 [file NA-007-D5NA00520E-s001.pdf]

Supplementary Information (SI) for Nanoscale Advances.
This journal is © The Royal Society of Chemistry 2025

SUPPORTING INFORMATION

Magnetoelectric/piezoelectric-based materials for a coupled electrical and mechanical stimulation for bone repair: an in silico study

Ilaria Faricelli[1,2], Martina Lenzuni[1]*, Paolo Giannoni[3], Paolo Ravazzani[4], Alessandra Marrella[1]

[1] Institute of Electronics, Computer and Telecommunication Engineering (IEIIT), National Research Council of Italy (CNR), Genoa, Italy

[2] Department of Informatics, Bioengineering, Robotics and Systems Engineering (DIBRIS), University of Genoa, Genoa, Italy

[3] Department of Experimental Medicine, Biology Section, University of Genoa, Genoa, Italy

[4] Institute of Electronics, Computer and Telecommunication Engineering (IEIIT), National Research Council of Italy (CNR), Milan, Italy

* Correspondence: martina.lenzuni@cnr.it

Table S1. *Materials properties used in COMSOL Multiphysics environment for cobalt ferrite (CFO), barium titanate (BTO), alginate gel matrix (ALG), hydroxyapatite (HAP) and cell (CELL) domains.*

Domain	Designation	Value	Unit	Reference
**CFO**	Density	5200	Kg/m^3	[1]
	Relative permittivity	10	-	[2]
	Electrical conductivity	10^{-5}	S/m	[3,4]
	Poisson ratio	0.48	-	[1]
	Young's modulus	$230*10^9$	Pa	[1]
	Magnetic susceptibility	0.42	-	Provided by manufacturer's measurements
	Magnetic saturation	$7.7*10^4$	A/m	Provided by

				manufacturer's measurements
	Saturation magnetostriction	-200	ppm	[1]
**BTO**	Density	5700	Kg/m^3	COMSOL library
	Relative permittivity	{1115.1, 1115.1, 1251.3}	-	COMSOL library
	Electrical conductivity	178.5	S/m	COMSOL library
	Elasticity matrix, Voigt notation	{150.4; 65.6; 150.4; 65.; 65.9; 145.5; 0; 0; 0; 43.9; 0; 0; 0; 0; 43.9; 0; 0; 0; 0; 0; 42.4}	GPa	COMSOL library
	Coupling matrix, Voigt notation	{0; 0; -4.32; 0; 0; -4.32; 0; 0; 17.4; 0; 11.4; 0; 11.4; 0; 0; 0; 0; 0}	C/m^2	COMSOL library
**ALG**	Density	1060	Kg/m^3	[5]
	Relative permittivity	80	-	[6]
	Electrical conductivity	0.02	S/m	[7]
	Poisson ratio	0.5	-	[5,8]
	Young's modulus	10*10^3	Pa	[9–11]
**HAP**	Density	3160	Kg/m^3	[12]
	Relative permittivity	77.61	-	[13]
	Electrical conductivity	3.7*10^{-11}	S/m	[14]
	Poisson ratio	0.3	-	[15]
	Young's modulus	102*10^9	Pa	[15]
	Elasticity matrix, Voigt notation	{117.6, 34.6, 117.6, 72.0, 72.0, 162.5, 0, 0, 0, 44.6, 0, 0, 0, 0, 44.6, 0, 0, 0, 0, 0, 41.5}	GPa	[15]
	Coupling matrix, Voigt notation	{0, 0, 15.06e-05, 0, 0, 15.06e-05, 0, 0, 18.6e-05, 177.66e-05, 55.53e-05, 0, 55.53e-05, -177.66e-05, 0, 0, 0, 0}	C/m^2	[16]
**CELL**	Density	1500	Kg/m^3	[17]
	Relative permittivity	80	-	[18]
	Electrical conductivity	0.25	S/m	[18,19]
	Poisson ratio	0.3	-	[17,20]
	Young's modulus	5.2*10^4	Pa	[21]

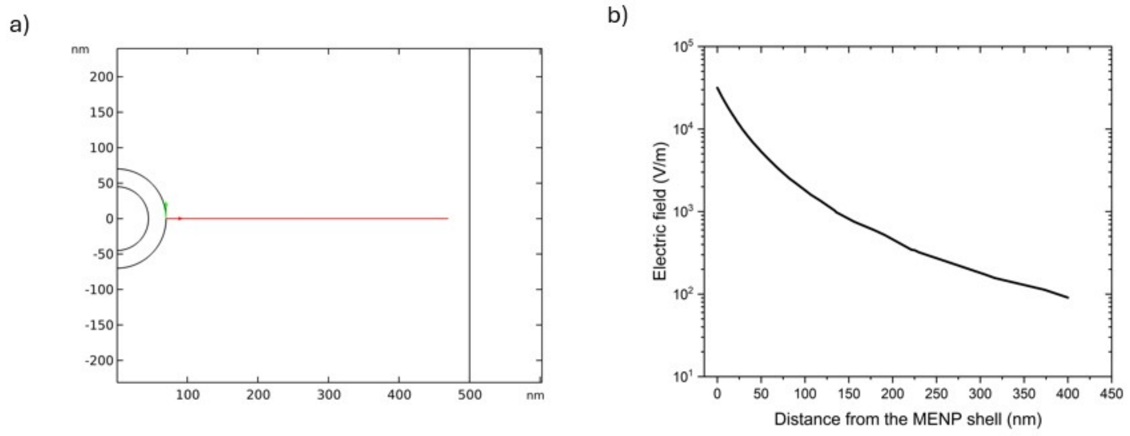

Figure S1. *Electric field distribution on the alginate gel matrix. (a) Horizontal cutline of 400 nm from the MENP shell and (b) the corresponding electric field values.*

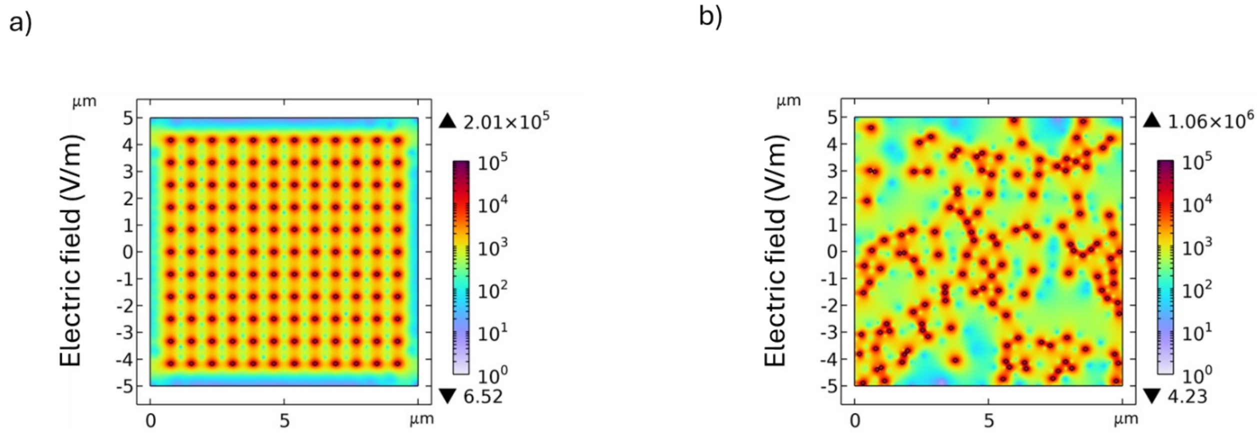

Figure S2. *Uniformly and randomly distributed MENPs. Figures (a) and (b) show the electric field distribution in the alginate gel matrix at MENP concentrations of 2% with homogeneous and random distributions, respectively.*

Table S2. *Percentage of alginate gel matrix area covered by various electric field (E) thresholds at 2% MENPs for uniform and random MENP distributions, with the corresponding estimated 3D MENPs volume fraction.*

**MENPs distribution**	**Estimated MENPs volume fraction (%)**	**MENPs coverage area (%)**	**Electric field threshold (V/m)**				
			**E > 10**	**E > 10^2**	**E > 10^3**	**E > 10^4**	**E > 10^5**
**Uniform**	**0.23**	**2.0**	99.98%	99.58%	91.32%	36.52%	0.93%
**Random**	**0.23**	**2.0**	99.98%	99.60%	89.06%	40.48%	1.08%

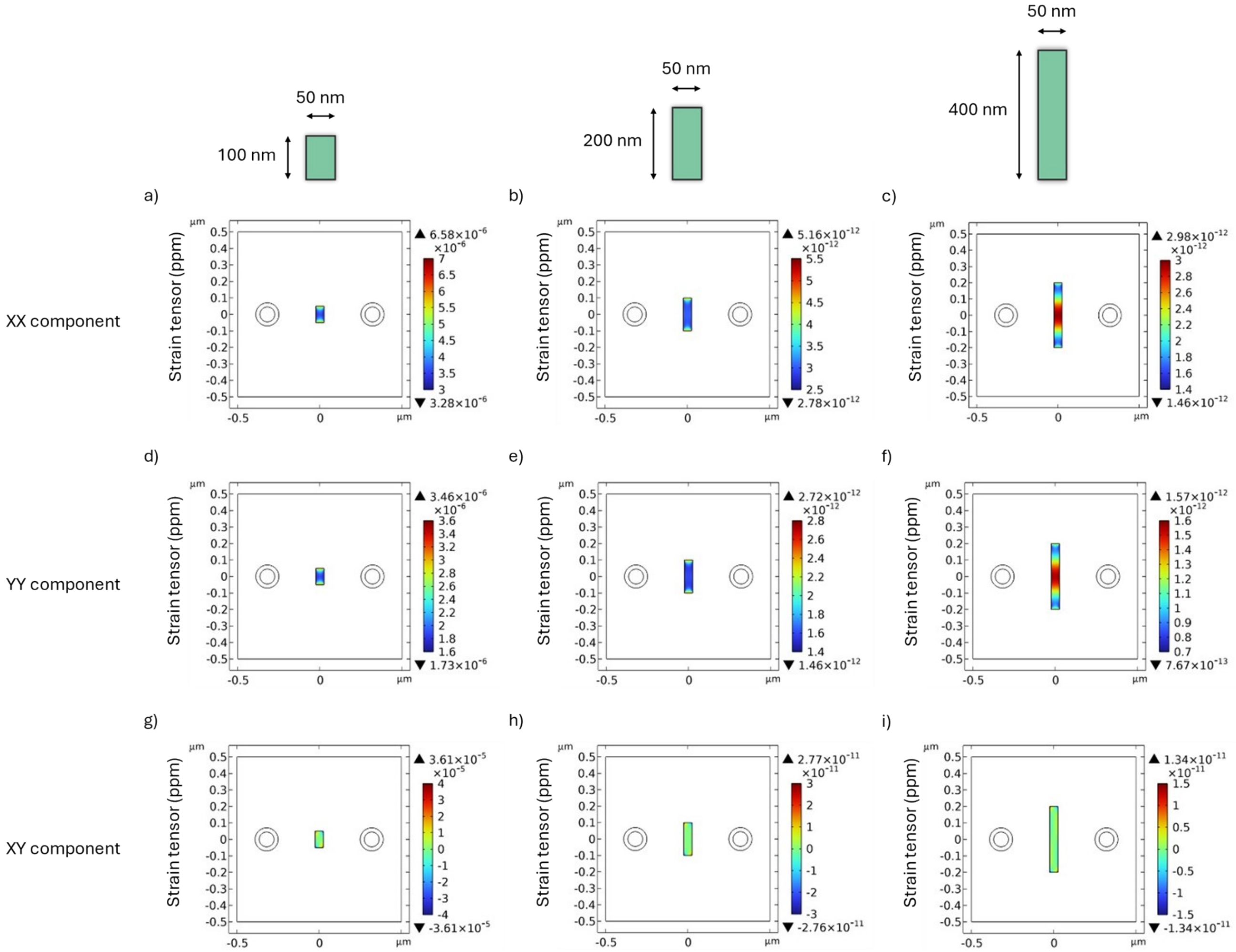

Figure S3. *Piezoelectric strain tensor distribution of HAP particles of varying sizes embedded in an alginate gel matrix between two MENPs under a DC magnetic field of 300 mT. The figures illustrate the piezoelectric strain tensor components XX (a–c), YY (d–f), and XY (g–i) for HAP particles with a fixed width of 50 nm and lengths of 100 nm, 200 nm, and 400 nm, respectively.*

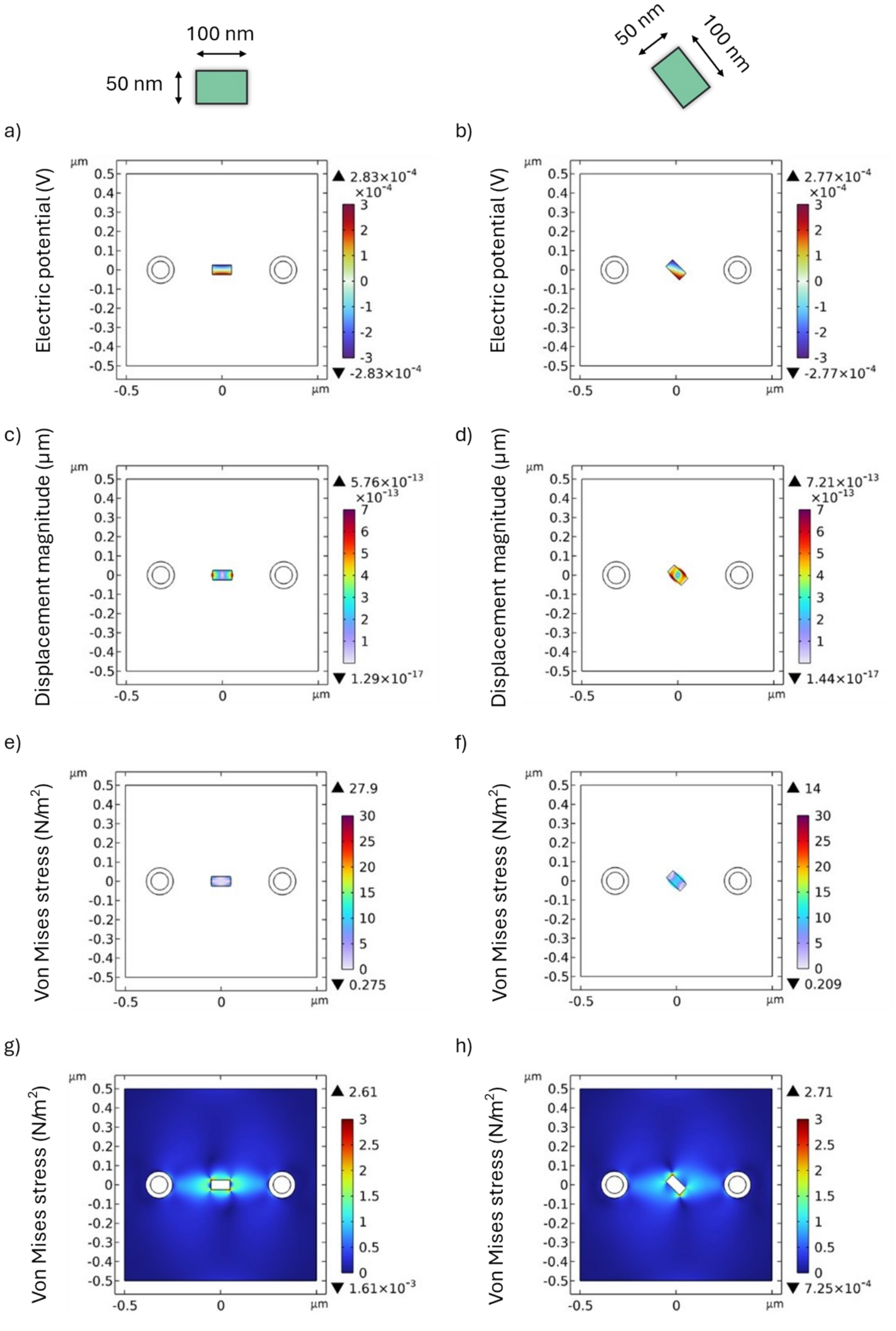

Figure S4. *Behavior of HAP particles of different orientations positioned between two MENPs in an alginate gel matrix under a static magnetic field. HAP particles with a length of 100 nm and varying orientations were placed between two MENPs at a concentration of 3% within the alginate gel matrix. Figures (a) and (b) depict the electric potential generated on the HAP particles oriented horizontally and partially rotated by 45 degrees, respectively. The corresponding displacement fields are shown in figures (c) and (d), while the von Mises stress distributions on the HAP particles are presented in figures (e) and (f). The von Mises stress induced in the alginate gel matrix is illustrated in figures (g) and (h).*

References

1 S. Fiocchi, E. Chiaramello, A. Marrella, G. Suarato, M. Bonato, M. Parazzini and P. Ravazzani, *PLOS ONE*, 2022, **17**, 1–17.
2 F. E. Carvalho, L. V. Lemos, A. C. C. Migliano, J. P. B. Machado and R. C. Pullar, *Ceramics International*, 2018, **44**, 915–921.
3 E. V. Gopalan, P. A. Joy, I. A. Al-Omari, D. S. Kumar, Y. Yoshida and M. R. Anantharaman, *Journal of Alloys and Compounds*, 2009, **485**, 711–717.
4 I. C. Nlebedim and D. C. Jiles, *Smart Materials and Structures*, 2014, **24**, 025006.
5 G. Kaklamani, D. Cheneler, L. M. Grover, M. J. Adams and J. Bowen, *Journal of the Mechanical Behavior of Biomedical Materials*, 2014, **36**, 135–142.
6 M. Esch, V. L. Sukhorukov, M. Kürschner and U. Zimmermann, *Biopolymers*, 1999, **50**, 227–237.
7 Z. Alves, N. M. Ferreira, S. Mendo, P. Ferreira and C. Nunes, *International Journal of Molecular Sciences*, 2021, **22**, 9943.
8 M. Ahearne, Y. Yang, A. J. El Haj, K. Y. Then and K.-K. Liu, *Journal of The Royal Society Interface*, 2005, **2**, 455–463.
9 J. Candiello, S. S. Singh, K. Task, P. N. Kumta and I. Banerjee, *Journal of Biological Engineering*, 2013, **7**, 9.
10 P. Harder, L. Funke, J. T. Reh, O. Lieleg and B. Özkale, *ACS Applied Materials & Interfaces*, 2025, **17**, 13513–13526.
11 X. Li, J. Gou, C. Maiti and O. J. Ilegbusi, *International Journal of Smart and Nano Materials*, 2024, **15**, 593–609.
12 J. Utomo, L. I. Noerjannah and N. Z. Rohmah, *Materials Today: Proceedings*, 2021, **44**, 3263–3267.
13 V. K. Kaliannagounder, N. P. M. J. Raj, A. R. Unnithan, J. Park, S. S. Park, S.-J. Kim, C. H. Park, C. S. Kim and A. R. K. Sasikala, *Nano Energy*, 2021, **85**, 105901.
14 B. Singh, S. Kumar, B. Basu and R. Gupta, *International Journal of Applied Ceramic Technology*, 2015, **12**, 319–328.
15 O. A. Osuchukwu, A. Salihi, I. Abdullahi, D. O. Obada, S. A. Abolade, A. Akande, S. Csaki and D. Dodoo-Arhin, *Data in Brief*, 2023, **48**, 109075.
16 V. Duarte, Y. González and M. Cerrolaza, *International Journal of Biomedical Engineering and Technology*, 2011, **5**, 211–228.
17 L. Wang, H.-Y. Hsu, X. Li and C. J. Xian, *BioMed research international*, 2016, **1**, 2735091.
18 A. Kaushik, R. Nikkhah-Moshaie, R. Sinha, V. Bhardwaj, V. Atluri, R. D. Jayant, A. Yndart, B. Kateb, N. Pala and M. Nair, *Scientific Reports*, 2017, **7**, 45663.
19 A. Ismail, M. Hughes, H. Mulhall, R. Oreffo and F. Labeed, *Journal of Tissue Engineering and Regenerative Medicine*, 2015, **9**, 162–168.
20 M. Pilia, T. Guda, S. M. Shiels and M. R. Appleford, *Journal of Biological Engineering*, 2013, **7**, 23.
21 J. Wang, M. Liu, Y. Shen, J. Sun, Z. Shao and D. M. Czajkowsky, *International Journal of Molecular Sciences*, 2018, **19**, 960.
